# Exposure of *Aspergillus flavus* NRRL 3357 to the Environmental Toxin, 2,3,7,8-Tetrachlorinated Dibenzo-*p*-Dioxin, Results in a Hyper Aflatoxicogenic Phenotype: A Possible Role for Caleosin/Peroxygenase (AfPXG)

**DOI:** 10.3389/fmicb.2019.02338

**Published:** 2019-10-15

**Authors:** Abdulsamie Hanano, Ibrahem Almousally, Mouhnad Shaban

**Affiliations:** Department of Molecular Biology and Biotechnology, Atomic Energy Commission of Syria, Damascus, Syria

**Keywords:** dioxin, aflatoxicogenicity, aflatoxin, caleosin, peroxygenase

## Abstract

Aflatoxins (AFs) as potent food contaminants are highly detrimental to human and animal health. The production of such biological toxins is influenced by environmental factors including pollutants, such as dioxins. Here, we report the biological feedback of an active AF-producer strain of *A. flavus* upon *in vitro* exposure to the most toxic congener of dioxins, the 2,3,7,8-tetrachlorinated dibenzo-*p*-dioxin (TCDD). The phenotype of TCDD-exposed *A. flavus* was typified by a severe limitation in vegetative growth, activation of conidia formation and a significant boost in AF production. Furthermore, the level of reactive oxygen species (ROS) in fungal protoplast was increased (3.1- to 3.8-fold) in response to TCDD exposure at 10 and 50 ng mL^–1^, respectively. In parallel, superoxide dismutase (SOD) and catalase (CAT) activities were, respectively, increased by a factor of 2 and 3. In contrast to controls, transcript, protein and enzymatic activity of caleosin/peroxygenase (AfPXG) was also significantly induced in TCDD-exposed fungi. Subsequently, fungal cells accumulated fivefold more lipid droplets (LDs) than controls. Moreover, the TCDD-exposed fungi exhibited twofold higher levels of AFB_1_. Interestingly, TCDD-induced hyperaflatoxicogenicity was drastically abolished in the AfPXG-silencing strain of *A. flavus*, suggesting a role for AfPXG in fungal response to TCDD. Finally, TCDD-exposed fungi showed an increased *in vitro* virulence in terms of sporulation and AF production. The data highlight the possible effects of dioxin on aflatoxicogenicity of *A. flavus* and suggest therefore that attention should be paid in particular to the potential consequences of climate change on global food safety.

## Introduction

Aflatoxins (AFs) are fungal lipid-derived toxins that provoke both acute and chronic toxicity in humans and animals. These toxins are produced by certain ascomycete fungi, most notably *Aspergillus flavus* and *Aspergillus parasiticus* (Yu et al., 2004; Shephard, 2008; Yu, 2012) and contaminate a range of fresh and stored food/feed products, therefore causing serious health, economic and ecological troubles. Thus, AFs were evaluated by the International Agency for Research on Cancer (IARC) as Group-1 agents (IARC, 2002). Of these, aflatoxin B_1_ (AFB_1_) is considered as the most potent carcinogen contaminant identified to date, with hepatocellular carcinoma as a major risk factor (Yu et al., 2004; Yu, 2012).

From an ecological point of view, *A. flavus* is widely spread in different niches where environmental factors play crucial roles in the phenotyping of fungal aflatoxicogenicity. More particularly, fungal spores have a remarkable connection with soil where its physicochemical and biological properties are determinant modulators of conidia biogenesis. Interestingly, increasing attention has been recently paid to the possible adverse effects caused by climate change in connection with fungal aggressivity (Medina et al., 2014; Assuncao et al., 2018). As a possible consequence of climate change, persistent environmental pollutants could dramatically increase because of the increased incidence of large-scale forest fires that has occurred over the last decade. Such pollutants, namely polychlorinated dibenzo-*p*-dioxins (PCDDs) and polychlorinated dibenzofurans (PCDFs), are considered the most toxic group of Persistent Organic Pollutants (POPs) (WHO, 2016). Due to their physicochemical properties, dioxins can persist in the environment and bioaccumulate in the organisms of a given ecosystem, including bacteria, fungi, plants, animals, and humans (Field and Sierra-Alvarez, 2008; Ishida et al., 2010; Anasonye et al., 2014; Hanano et al., 2014b).

The cytotoxicity of dioxins is expressed through their high lipophilicity which exercises a force driving on dioxins toward the cellular lipids, affecting therefore their metabolism and functions (Lawrence and Kerkvliet, 1998; Hanano et al., 2014a, b, 2018b; Cranmer-Byng et al., 2015). In connection with this, dioxin-induced alternations in lipid metabolism and oxidative status could have important feedback effects on the biology of AF-producing fungi in general and on their aflatoxicogenicity in particular. This is because aflatoxins are ultimately synthesized from acetyl-CoA via fatty acid polyketide under an excessive oxidative status (Reverberi et al., 2010; Fountain et al., 2018; Kenne et al., 2018). Beyond the modulatory role of reactive oxygen species (ROS), fungal lipids and their metabolites, more particularly a class of them know as oxylipins, can play as modulators of AF biosynthesis (Roze et al., 2007; Gao and Kolomiets, 2009; Fountain et al., 2014). The fungal oxylipin-biosynthesizing enzyme, the AfPXG, has been recently characterized as a caleosin with a peroxygenase activity and therefore referred to as Caleosin/Peroxygenase (Hanano et al., 2015). This AfPXG is necessary for fungal growth, development and AF production (Hanano et al., 2015). More recently, we presented detailed genetic, molecular and biochemical evidence on the direct implication of AfPXG in the biosynthesis of aflatoxins and their trafficking and extracellular secretion via lipid droplets (LDs) (Hanano et al., 2018a). A similar implication of fungal LDs in the sequestration and trafficking of lipid-soluble molecules has been also suggested in plants. The LDs isolated from oilseeds or date palm stones can sequester *in vitro* a variety of hydrophobic organic contaminants, with the greatest activity found for dioxins (Boucher et al., 2008; Hanano et al., 2016a). Likewise, the dioxin-sequestration activity of LDs was also demonstrated *in planta*, where the majority of experimentally administrated dioxins to Arabidopsis or date palm seedlings was found within LD fractions (Hanano et al., 2016b, 2018b). This high activity was based on the production of numerous small LDs, and this ensures the highest contact surface between the LDs and the dioxins (Hanano et al., 2016b, 2018b,c).

Based on the above, hydrophobic pollutants could have significant effects on the biology of AF-producing fungi and subsequently on their aflatoxicogenicity. To that end, we *in vitro* exposed the *A. flavus* NRRL 3357 to 2,3,7,8-tetrachloronated dibenzo-*p*-dioxin (TCDD), the most toxic congener of the dioxins group. Then, the toxicological effects of dioxins were characterized in terms of fungal growth, development and aflatoxin production. In a particular connection with the TCDD-induced accumulation of LDs, the transcripts and enzymatic activity of the caleosin/peroxygenase AfPXG were discussed using the wild-type (WT) and the AfPXG-silenced lines of *A. flavus*. Finally, the virulence of the TCDD-exposed *A. flavus* was assayed. This work highlights the biological effects on the aflatoxicogenicity of *A. flavus* following its exposure to the persistent environmental pollutant, the dioxin.

## Materials and Methods

### Materials, Chemicals, Strains, Culture Conditions, and Treatments

Oligonucleotides were purchased from either Eurofins or Sigma-France. Aniline, cumene hydroperoxide, aflatoxin AFB_1_ and all organic solvents were purchased from Sigma-Aldrich, Germany. The *A. flavus* strain NRRL3357 was supplied from the Faculty of Agricultural Sciences, Gembloux, Belgium. Stock cultures of *A. flavus* were maintained in slant tubes at 4°C on potato dextrose agar (PDA) (Difco Laboratories, United States). For solid or liquid cultures of *A. flavus*, stock cultures were transferred onto Petri dishes containing PDA or into a 500-mL Erlenmeyer flask containing 100 mL of PD broth and kept for 7 days at 28°C.

### Biomass and Conidia Number Measurements

Fungal biomass, expressed as dry weight per plate, was measured as previously described (Hanano et al., 2015). In parallel, the total conidia fraction for each plate was harvested and taken up in 5 ml solution of 0.01% Tween 80, diluted to 1:10, and counted using a hemocytometer.

### Preparation of *A. flavus* LDs Fraction and Peroxygenase Activities Assay

Isolation of fungal LD fractions was performed essentially as described by Ferreira de Oliveira and co-workers (Record et al., 1998; Ferreira De Oliveira et al., 2010) with brief modification as described previously (Hanano et al., 2015). In brief, 5 g of fungal mycelium was ground into a mortar in the presence of liquid nitrogen until a fine powder was obtained. The dried powder was immediately hydrated with 10 mL of buffer A (100 mM potassium pyrophosphate, 0.1 M sucrose and pH 7.4). The mixture was then gently homogenized for 5 min using an ultra-dispenser (T25 digital ULTRA-TURRAX, IKA laboratory, Germany) and centrifuged for 10 min at 10,000 × *g*. The resulting supernatant was centrifuged at 100,000 × *g* for 1 h, and this enabled the obtainment of a floating white pad layer consisting of LDs. LDs were gently collected from the top of the tube using a Pasteur pipette, then carefully washed twice with 5 mL of buffer B (buffer A without sucrose). After a final centrifugation (100,000 × *g* for 1 h), the LD fraction was suspended in 1 mL of buffer B and stored at 4°C for further analysis. Peroxygenase activity was assayed by oxygenation of aniline as a substrate (Blee and Durst, 1987; Hanano et al., 2006).

### Analysis of LDs

Microscopic imaging was performed at a magnification of 40 × under a LEICA MPS60 microscope using an Olympus FE-4000 camera. The purity of LD preparation, their native encapsulation and their number per mL were evaluated under a LEICA MPS60 light microscope, and the images were taken at a magnification of 40 × immediately after preparation.

### SDS–PAGE and Western Blotting

Lipid droplet-associated proteins were isolated according to Katavic et al. (2006) and analyzed by SDS–PAGE using 12% polyacrylamide gels and electroblotted onto a PVDF membrane (Millipore) in a Semi-Dry Transfer Cell (Bio-Rad). Caleosins were immunodetected by incubating the membrane with a polyclonal antibody prepared from the complete sequence of the CLO1 caleosin isoform from *Arabidopsis thaliana*, as described previously (Hanano et al., 2018c).

### Genes, Primers and Transcript Analysis

Nucleotide sequences of primers used in this section are listed in Table 1. For the gene expression studies, *A. flavus* was grown as described previously. Total fungal biomass was collected for total RNA isolation using an RNeasy kit according to the manufacturer’s instructions (Qiagen, Germany). DNA traces were removed by 2 units of RNase-free RQI DNase (Promega, United States) for 1 h at 37°C. RNAs were diluted to 50 ng μL^–1^ using RNase-free water and stored at −80°C. cDNA synthesis was performed using M-MLV RT (Invitrogen) as described previously (Hanano et al., 2015). Real-time PCR was carried out in 96-well plates using an AriaMx Real-time PCR System from Agilent technologies, United States. The 25-μl reaction mixtures contained 0.5 mM of each target and reference gene primers, 12.5 μl of SYBR Green qRT-PCR mix (Bio-Rad, United States) and 2.5 μl of 10-fold diluted cDNA. qRT-PCR conditions were as previously described (Hanano et al., 2015). Each point was triplicated and the average of *C*_*T*_ was taken. The relative quantification RQ = 2^(–ΔΔ*DD*)^ of the target gene was calculated.

**TABLE 1:** Primers used for the transcriptional analysis of AF-biosynthesis cluster genes.

**Gene**	**Primers name**	**Nucleotide sequence (5′–3′)**	**Primer position**	**Amplicon (bp)**
*fas-a*	*fasaF fasaR*	CAACGCCAACGCTATTCGAG GTAATGCCACACGATTCGGC	537–556 697–716	180
*fas-b*	*fasbF fasbR*	ATCCACTCGACATCATCGCC TTGATGTCACGTCGGCTGAA	2468–2487 2563–2582	115
*pksA*	*pksAF pksAR*	TAGTGTGCCTCTGCCAGTTG GGAACCCATGCAGAATCCCA	254–273 341–360	107
*nor-1*	*nor1F nor1R*	GCATCGGACGAGGTCTCATT CTGGGCATCAGTTTCCGAGT	158–177 308–327	170
*norA*	*norAF norAR*	TTGGTACTGAGCGAGGAGGA TTCTAGCCGAGTGTTGCAGG	940–959 1079–1098	159
*avnA*	*avnAF avnAR*	ATCGACGACTGTTGGCCTTT CGAGTCTCCAAAAGCGAGGT	386–405 554–573	188
*adhA*	*adhAF adhAR*	TCTAGAGACGGGGCAGAACA TGCAAAGGAGACACCTGCAA	147–166 299–318	172
*avfA*	*avfAF avfAR*	AGTACCGGCCTTCGTTCATC AGTCTGTAGCCCGTTGGTTG	411–430 568–587	177
*estA*	*estAF estAR*	ACGCTACGAGATGATGCCAG TCCCCCGAAGAAAGTCCTCT	766–785 896–915	150
*vbs*	*vbsF vbsR*	CCGCTCTGATGACTCCCTTC GTCCGATGCAACAATCTCGC	1509–1528 1647–1666	158
*verb*	*verbF verbR*	GATGCTCAATAACGCTGCCG GTAAGGTACGGCAGATGCGA	963–982 1129–1148	186
*ver-1*	*ver1F ver1R*	TGGTGAACTACGCCCATTCC CACCGTCTCCGCCATTAACT	110–129 227–246	137
*verA*	*verAF verAR*	CCTCAGCAGCCACCCAAATA CCGCCACTTCTTCCAAGTCT	290–309 398–417	128
*omtB*	*omtBF omtBR*	GCAAACGGCAAATTCAGGGT CGCTAGAGTTATCGGCGTGT	71–90 224–243	173
*omtA*	*omtAF omtAR*	ATGTGACGAAGTGATGCGGT CTCGCATTTCAGCTGCGTTC	294–313 430–449	156
*ordA*	*ordAF ordAR*	ATTTGTGTTCGGCTTTGGGC TGGGCGAGATGAAGAAGCAG	1524–1543 1611–163	107
*apa-2*	*apa2F apa2R*	CGCTATTGCTGCTTTTCGCT GCATCGGTAGCCCTCTTGTT	691–710 829–848	158
*TE*^a^	*TEF TER*	AGGCTTTCTTTGTGAGCCGT ATAGCTGATGCTGACGGAGC	357–376 469–488	132

*^*a*^TE – Transcriptional Enhancer encoding gene.*

### Extraction, Clean-Up, TLC and HPLC Analysis of Aflatoxin

The extraction of AF was done according to Bertuzzi et al. (2011) using 50 mL of chloroform for 1 h on a rotary-shaker, and extracts were purified as described previously (Shannon et al., 1983). The extracted AF samples were analyzed by thin layer chromatography (TLC) using a C_18_ reversed-phase TLC plate (aluminum sheets measuring 20 × 20 cm with 200-μm layers, Merck, Germany) and the chromatogram was developed using a solvent system of chloroform/acetone (90:10, v/v). After development, the spot with the same *Rf*-value as the AFB_1_ standard was scraped off, re-extracted with chloroform and evaporated to dryness under nitrogen. The extract was taken up in 100 μL acetonitrile in an amber-colored vial under refrigeration. AF was analyzed using a Jasco LC-2000 plus series HPLC system (Jasco, United States) with a fluorescence detector (RF-10Axl, Shimadzu) (λexc 247 nm; λem 480 nm) and a C18 column (Eclipse XDB-C18 150 × 4.6 mm, 5 μm; Agilent, United States, column temperature 35°C) as described previously (Hanano et al., 2015).

### Detection and Quantification of ROS in Fungal Tissue

The accumulation of ROS was detected in fungal spores protoplast using a 2′,7′-dichlorofluorescin diacetate (DCFH-DA) staining protocol (Chang et al., 2011). The DCFH-DA, a cell-permeable non-fluorescent probe that turns to highly fluorescent 2′,7′-dichlorofluorescein in the presence of ROS (Sigma-Aldrich, United States) was dissolved into dimethyl sulfoxide (DMSO), and the stock solution was conserved at −20°C. Fungal protoplasts, previously washed with sterile deionized water, were incubated into 1 mL of freshly prepared solution of 10 μM DCFH-DA in phosphate-buffered saline (PBS) for 4 h at 28°C in the dark. Later, the protoplasts were collected by a brief centrifugation and washed twice with PBS and subsequently examined under a Nikon Eclipse Ti-U fluorescent microscope. Micrographs were taken at a magnification of 10 × using a Nikon Ti-U camera. The fluorescence intensity was analyzed using the software Image-Pro Plus 6.0.

### SOD and CAT Enzymatic Activities

Preparation for enzyme activities was carried out as described previously (Zhang et al., 2012) with some modification. Briefly, 5 g of fungal tissues was homogenized with 5 mL potassium phosphate buffer (pH 7.5) containing 1 mM ethylenediaminetetraacetic acid (EDTA), 3 mM DL-dithiothreitol and 5% (w/v) insoluble PVPP on ice. Subsequently, the homogenate was centrifuged at 12.000 rpm for 5 min, and the supernatant was therefore used for analyzing the enzymatic activities. Superoxide dismutase (SOD) activity was assayed by measuring its ability to inhibit the photochemical reduction of nitroblue tetrazolium (NBT) as described by Beauchamp and Fridovich (Beauchamp and Fridovich, 1971). Catalase (CAT) activity was measured by the method of Azevedo et al. (1998). Activity was determined by monitoring the decrease in absorbance due to H_2_O_2_ reduction at 240 nm for 2 min.

### siRNA-Silencing of AfPXG Gene

Small interference RNAs (siRNA) design primers, preparation of fungal protoplast and siRNA delivery were performed according to Hanano et al. (2018a). In brief, delivery of siRNA to protoplasts was done in sterile 1.5 mL tubes. A total of 10 μL of siRNA primer (100 nM) was mixed with an equal volume of Lipofectin reagent (Invitrogen Life Technologies, United Kingdom) and kept for 15 min at 20°C. A volume of 50 μL of protoplasts was added, gently mixed and incubated at 20°C for 24 h to allow transfection to proceed. The transfected protoplasts were therefore inoculated in 10 mL of PD medium with 1.2 M of sorbitol for 7 days at 28°C in the dark. All experiments were performed using three biological replicates.

### Inoculation of Maize Seeds With *A. flavus* and Biomass Estimation

A quantity of 100 g of maize (*Zea mays*) was sterilized by immersion into 70% ethanol for 1 min. After drying, grains were placed in a sterile petri plate and directly inoculated with 200 μl of liquid culture of *A. flavus* in PD broth. Inoculated grains were incubated at 28°C for 7 days. Fungal biomass was estimated on day 7 by careful washing of infected grains, filtration and then weighing the fungal growth expressed in grams of fresh weight per 100 g of grains.

### Statistical Analysis

All data presented are expressed as means ± standard deviation (SD). Comparisons between control and treatments were evaluated by *t*-test. Difference from control was considered significant as *P* < 0.05, very significant as *P* < 0.01, and highly significant as *P* < 0.001.

## Results

### Characterization of the TCDD-Exposed Phenotype of *A. flavus*

**FIGURE 1:**
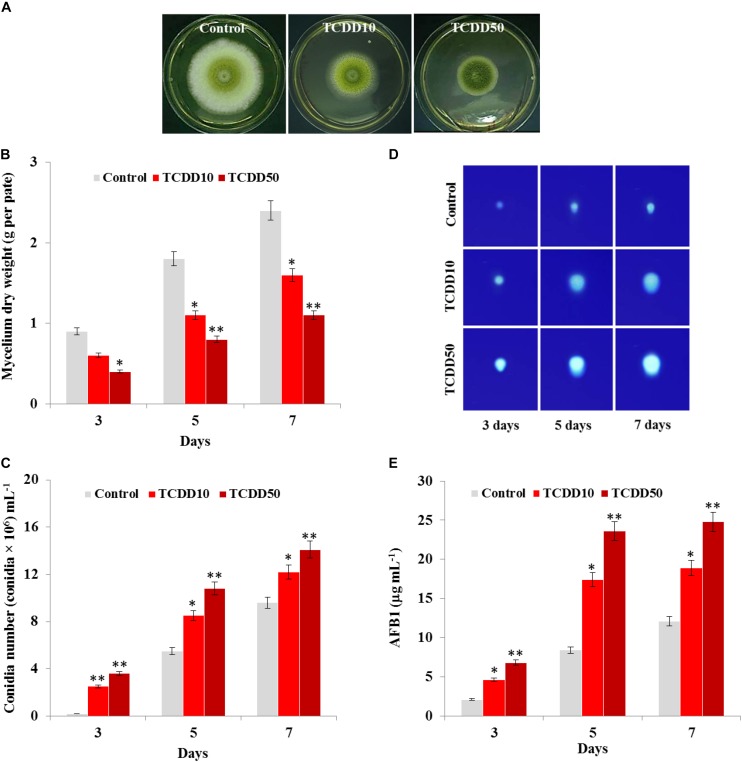
Characteristic of *A. flavus* TCDD-exposed phenotype. **(A)** Photographs of 5-day-old cultures of *A. flavus* on PDA-plates upon the exposure to TCDD at 10 and 50 ng L^–1^ that referred as to TCDD10 and TCDD50, respectively, compared with non-exposed fungus control. **(B,C)** Variation in fungal mycelium dry weight and spore number, respectively, as a function of the exposure to TCDD at both doses. **(D)** Sections of Thin Layer Chromatography (TLC) plate showing the blue-fluorescent spots under the UV light that correspond to the AFB_1_ secreted by the TCDD-exposed fungi compared to control. **(E)** Quantitative data of AFB_1_ as estimated by UV-detector HPLC. All measurements were done in triplicate and the presented data are means ± *SD* (*n* = 3). Difference between treatments and control was significant (^∗^*P* < 0.05) or very significant (^∗∗^*P* < 0.01) when analyzed by *t*-test.

The photographs presented in Figure 1A show that the TCDD-exposed *A. flavus* were phenotyped by limitation in fungal growth and activation of conidia formation compared to control. Inversely to control, the mycelium dry weight of fungi per plate was significantly reduced when they were exposed to TCDD and formed only about half of control growth at the highest dose of TCDD (50 ng L^–1^) on days 3, 5, and 7 after inoculation (Figure 1B). More surprisingly, the TCDD-exposed fungi tended to produce spores more actively compared to the control. For example, on day 7, while the fungus that was exposed to 50 ng L^–1^ produced more than 14 × 10^6^ mL^–1^, the control produced about 9.6 × 10^6^ mL^–1^ (Figure 1C). Furthermore, the blue fluorescent spot of AFB_1_ was increasingly detected on the TLC as a function of TCDD treatment (Figure 1D). Quantitatively speaking, the amount of AFB_1_, measured by HPLC, was doubled in the TCDD-exposed fungi compared to control, where the highest concentration of AFB_1_ (24.8 μg mL^–1^) was detected when *A. flavus* was exposed to 50 ng L^–1^ on day 7 after inoculation (Figure 1E). Together, these data suggest that dioxin exposure causes certain limitation in fungal growth and boosts conidiation and AFB_1_ production in *A. flavus*.

### The TCDD-Exposed Phenotype of *A. flavus* Has an Enhanced Level of Oxidative Status

**FIGURE 2:**
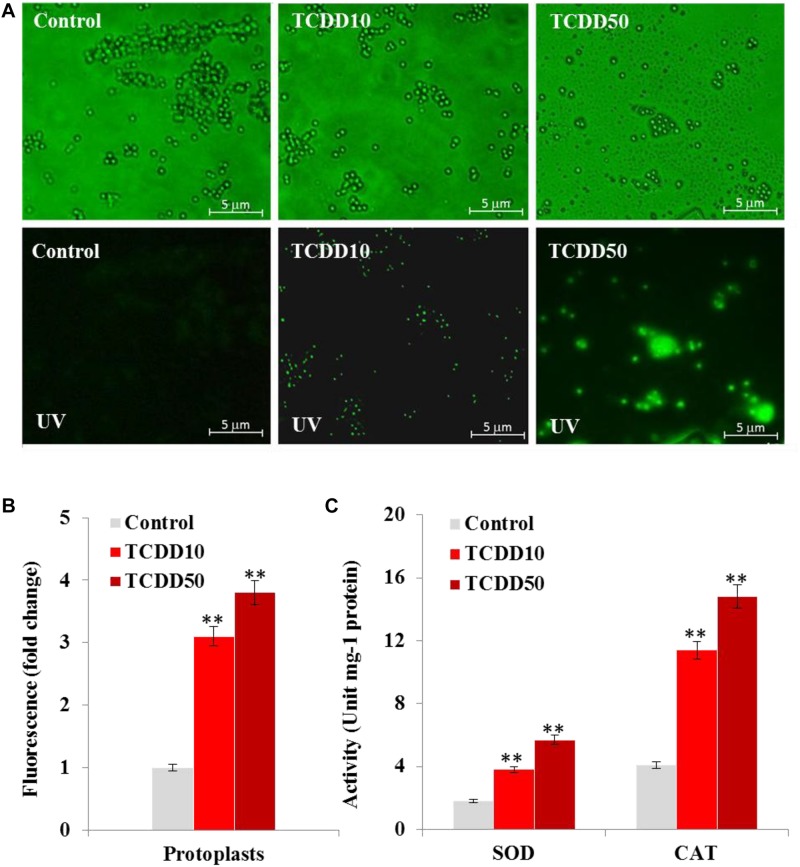
The exposure to TCDD enhances the accumulation of ROS in fungal tissues. **(A)** Micrographs of fungal spore protoplasts stained with DCFH-DA, a cell-permeable non-fluorescent probe that turns to highly fluorescent 2′,7′-dichlorofluorescein in the presence of cellular ROS. Samples were examined under a Nikon Eclipse Ti-U fluorescent microscope and micrographs recorded at a magnification of 10 × a using a Nikon Ti-U camera. Bar represents 5 μm. **(B)** The ROS fluorescence intensity was analyzed by software Image-Pro Plus 6.0. The fold change in fluorescence intensity was estimated by comparing the TCDD-treated samples for spore protoplasts with their respective controls that were considered as 1. Data were shown as the mean ± *SD*. **(C)** Enzymatic activities of SOD and CAT in fungal tissues in response to TCDD exposure at indicated concentrations. All measurements were done in triplicate and the presented data are means ± *SD* (*n* = 3). Difference between treatments and control was significant (^∗^*P* < 0.05) or very significant (^∗∗^*P* < 0.01) when analyzed by *t*-test.

It is well know that AF biosynthesis is naturally stimulated under stress oxidative status caused by a high level of ROS (Reverberi et al., 2008). Thus, the high production of AF by the TCDD-exposed *A. flavus* raises the question on whether such exposure will boost the accumulation of ROS in fungal tissues. To evaluate that, the accumulation of ROS in fungal spore protoplasts was detected using the DCFH-DA, a cell-permeable non-fluorescent probe that turns to highly fluorescent 2′,7′-dichlorofluorescein in the presence of cellular ROS. As the photographs show, the intensity of green fluorescence that was proportionally related to the amount of ROS was remarkably augmented in a response to the exposure to TCDD at 10 and 50 ng mL^–1^ in fungal spore protoplasts compared to their respective controls (Figure 2A). When the fluorescence intensity was analyzed by the software Image-Pro Plus 6.0, we stated that such intensity was higher – about 3.1- to 3.8-fold in protoplasts exposed to TCDD at 10 and 50 ng L^–1^, respectively, compared to their representative controls (Figure 2B). In connection with ROS level, we have measured the cellular enzymatic activities of SOD and CAT; both enzymes are activated by the excess ROS and react to neutralize the O^2.–^ and H_2_O_2_, respectively. SOD activity was stimulated by a factor of 2 and 3 in TCDD-exposed fungi to 10 and 50 ng mL^–1^, respectively, compared to controls (Figure 2C). Also, CAT activity was induced 2.7- to 3.6-fold in such treatments compared to controls (Figure 2C). These data suggest that the exposure to dioxin led to enhanced ROS accumulation in fungal tissue, and subsequently the ROS-scavenging enzymatic activities were induced.

### TCDD Induces the Expression of AfPXG, the Accumulation of LDs and Subsequently the Production of AFB_1_ in *A. flavus*

**FIGURE 3:**
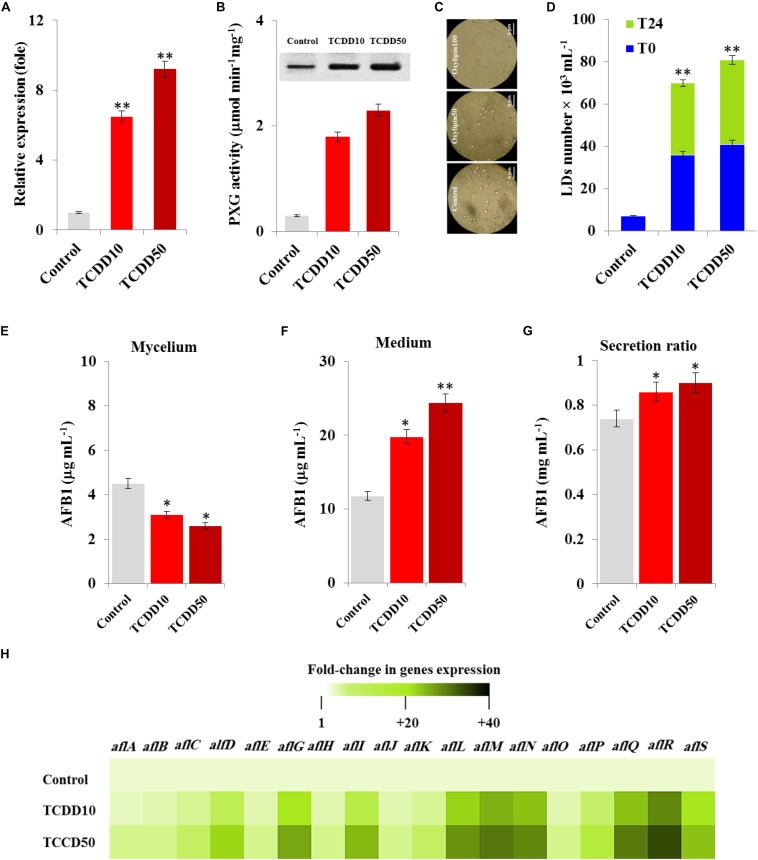
The TCDD induces the functional expression of AfPXG, the accumulation of LDs and the production of AfB1. **(A)** Transcripts level of AfPXG gene, evaluated by RT-qPCR, in the fungal tissues exposed or non-exposed to TCDD. **(B)** The peroxygenase activity of AfPXG, estimated by the hydroxylation of aniline at 310 nm. **(C)** Micrographs of LDs isolated from fungal tissues exposed or not exposed to TCDD. Samples were examined under a LEICA MPS60 light microscope and the images were taken at a magnification of 40 × immediately after preparation. Bar represents 5 μm. **(D)** LD number was estimated using a hemocytometer. **(E,F)** Measurement of AFB1 in the fungal mycelium and in the culture medium of *A. flavus* exposed or not exposed to TCDD. **(G)** Secretion ratio of AFB1 by fungi exposed or not exposed to TCDD (calculated as the ratio of AFB1 in the medium to AFB1 in medium plus mycelia in a 100 mL culture). **(H)** Relative quantification RQ = 2^(–ΔΔ*DD*)^ of AF-biosynthesis gene transcripts upon the exposure to TCDD compared to control. The color scale (white-green-black) indicates relative changes of transcripts of 1, +20, and +40-fold, respectively. For each gene, the expression level in the control was defined as 1, and the corresponding increase in gene transcript under treatments was calculated. All measurements were done in triplicate and the presented data are means ± *SD* (*n* = 3). The differences between control and treatments were significant when analyzed by *t*-test (^∗^*P* < 0.05; ^∗∗^*P* < 0.01).

We recently reported that the caleosin/peroxygenase AfPXG of *A. flavus* modulates the biosynthesis of AF and its trafficking via the LDs (Hanano et al., 2018a). Likewise, certain isoforms of plant caleosins/peroxygenase were strongly induced by dioxin (Hanano et al., 2018b). In connection with this, we were interested in gaining more insight into the biological implication of AfPXG in fungal responses to dioxin exposure. At transcription level, the expression of AfPXG was significantly induced upon exposure to TCDD. The transcript level of AfPXG was augmented 6.5- and 9.2-fold as a function of exposure to TCDD at 10 and 50 ng mL^–1^, respectively, compared with control (Figure 3A). This was synchronized with a similar increase in protein level of AfPXG as well as in its enzymatic activity, measured by hydroxylation of aniline, where this activity was increased from 0.3 in the control to 1.8 and 2.3 (μmol min^–1^mg^–1^) in the exposed fungi to 10 and 50 ng mL^–1^ of TCDD (Figure 3B). Subsequently, the TCDD-exposed fungi accumulated an increasing number of LDs as shown in the micrographs under light microscope (Figure 3C). Unlike the LDs isolated from control fungi, the number of LDs isolated from TCDD-exposed fungi was augmented by a factor of 5, and this number was stable 24 h after preparation compared with the critical stability of control LDs (Figure 3D). The increasing accumulation of LDs in the TCDD-exposed fungal tissues raises the question about their cellular activity in the trafficking and secretion of AF. Interestingly, despite the decreasing amount of AFB_1_ that detected in the TCDD-exposed fungal tissues (Figure 3E), these fungi secreted more AFB_1_ into the medium (Figure 3F) and this elevated their secretion ratio to about 0.9 versus 0.7 for the control (Figure 3G). The high production of AFB_1_ upon exposure to TCDD was effectively related with important increases in the transcripts level of certain key genes, e.g., *alfD*, *alfG*, *alfI*, *alfL*, *alfM*, *alfN*, *alfQ*, *alfR*, and *alfS* of the AF biosynthesis pathway (Figure 3H). These data suggest that the exposure of *A. flavus* to dioxin induces the expression of AfPXG, increases the accumulation of stable LDs and therefore enhances the production of AF.

### TCDD-Induced Hyperaflatoxicogenicity Is Reduced in AfPXG-Silencing Strain

**FIGURE 4:**
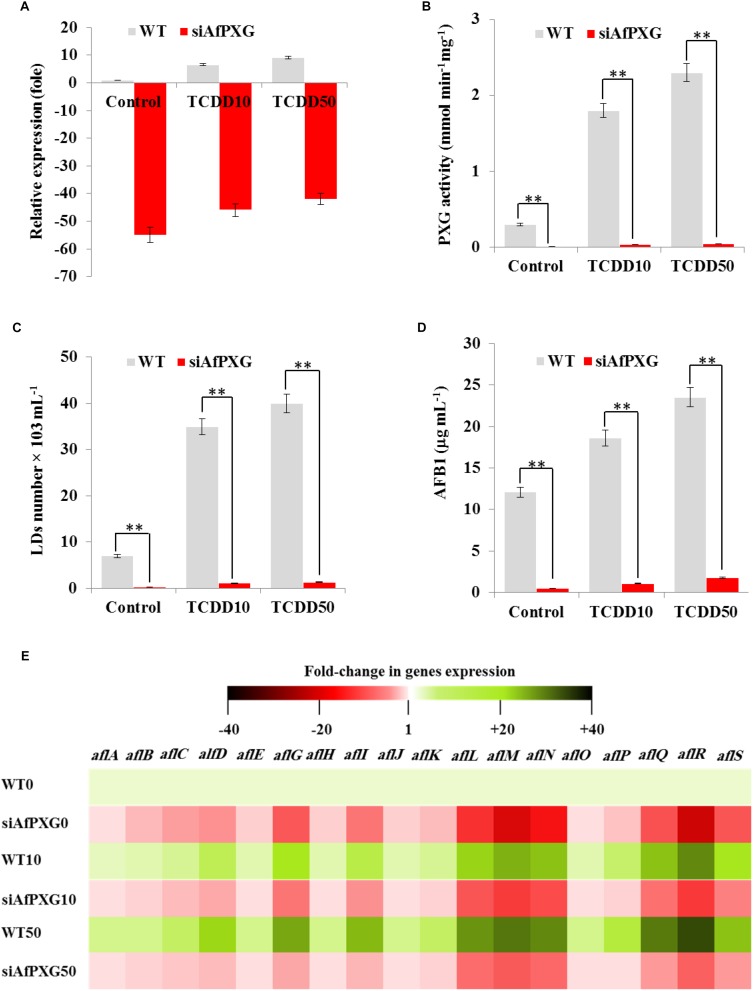
The TCDD-induced hyperaflatoxicogenic phenotype is abolished in the AfPXG-silencing strain. **(A)** Relative quantification of AfPXG transcripts in the AfPXG-silencing strain (siAfPXG) and the wild type (WT) upon their exposure to TCDD at the indicated concentrations. **(B)** Estimation of the peroxygenase activity in siAfPXG and WT after exposure to TCDD compared with their respective controls. The activity was estimated by the hydroxylation of aniline at 310 nm. **(C)** Number of LDs isolated from siAfPXG and WT after exposure to TCDD. **(D)** Concentration of AFB1 secreted by siAfPXG and WT upon their exposure to TCDD at both concentrations compared with their respective controls. **(E)** Transcripts levels of the AF-biosynthesis genes in fungal tissue of siAfPXG and WT after their exposure to TCDD at both concentrations. This was carried out using the RT-qPCR system. Three measurements were done in three cDNAs prepared from three individual fungal tissues for each line. The color scale (red-white-green) indicates positive changes of transcript abundance of - 40-, 1, and +40-fold, respectively. For each gene, the expression level in the control was defined as 1, and the corresponding abundance changes under treatments were calculated directly using the software installed in the Applied Biosystems qPCR system. The differences between treatments and control were significant when analyzed by *t*-test (^∗^*P* < 0.05; ^∗∗^*P* < 0.01).

To confirm the biological link between the TCDD-induced aflatoxicogenicity and the function of AfPXG, the TCDD was administrated to *A. flavus* WT as well as to AfPXG-silenced strain (referred as to siAfPXG) in which the AfPXG gene was silenced by about half using siRNAs (Hanano et al., 2015, 2018a). Our data indicated that the silencing of AfPXG was briefly restored upon treatment with TCDD, but its transcript level was still about 42- to 46-fold lower compared to the control (Figure 4A). In parallel, the silencing of AfPXG was also confirmed by the absence of peroxygenase activity in the control as well as in the TCDD-treated samples (Figure 4B). Furthermore, the treatment with TCDD, at both concentrations, did not lead to increased accumulation of LDs in the siAfPXG strain as it did in the WT (Figure 4C). Subsequently, the treatments with TCDD at both concentrations did not provoke an over-secretion of AFB_1_ in the siAfPXG strain as it did in the WT. For example, while the production of AFB_1_ in the WT treated with the highest dose of TCDD was highly stimulated to reach about 23.5 μg mL^–1^, this treatment failed to provoke a similar effect in the siAfPXG strain where its AFB_1_ production did not exceed 1.8 μg mL^–1^ (Figure 4D). Apparently, the failure of the TCDD-exposed siAfPXG strain in stimulating the AF production was related with a severe down-regulation of a set of key genes in AF-biosynthesis pathway. The heat map (Figure 4E) shows that upon its exposure to TCDD, the siAfPXG strain still had low levels of *alfB*, *alfC*, *alfD*, *alfG*, *alfI*, *alfL*, *alfM*, *alfN*, *alfQ*, *alfR*, and *alfS* transcripts compared with WT. Altogether, these results suggest that the TCDD-induced aflatoxicogenicity of *A. flavus* is likely mediated by AfPXG.

### The TCDD-Exposed *A. flavus* Shows More Aggressivity to Infect Maize Grains

**FIGURE 5:**
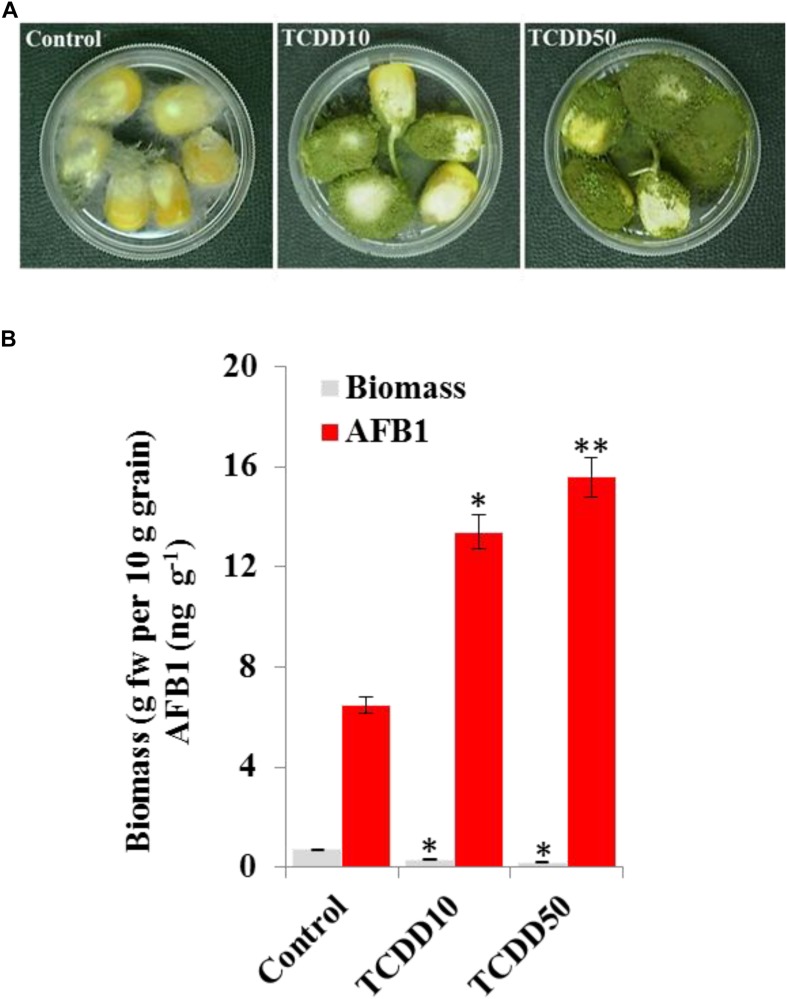
The exposure to TCDD boosts the aggressivity of *A. flavus* against the maize grains. **(A)** Photographs of infected maize grains with *A. flavus* previously exposed to TCDD at 10 and 50 ng L^–1^ referred to as TCDD10 and TCDD50, respectively, compared with non-exposed fungus control. After sterilizing, grains were placed in a sterile petri plate and directly inoculated with 200 μl of liquid culture of *A. flavus* in PD broth contaminated or not contaminated with TCDD. The photographs were taken 8 days after inoculation. **(B)** Estimation of fungal biomass and AFB1 production in the three samples. Asterisks indicate significant differences between treatments and control (^∗^*P* < 0.05; ^∗∗^*P* < 0.01).

The induced aggressivity of *A. flavus* caused by the exposure to TCDD was assayed *in vitro* using grains of maize. For that, the spores of *A. flavus* WT strain grown in contaminated medium with TCDD were collected and used to infect the grains. Then, the fungal biomass and AF production were evaluated and compared with control. The photographs of infected grains (Figure 5A) show a brief limitation in fungal growth but a more pronounced activity in conidiation of TCDD-exposed fungi compared to the non-exposed fungus. This was confirmed by the decreasing amount of fungal biomass, expressed as (g dry weight per 10 g of grains) in the TCDD-exposed fungi, at both concentrations (2.3 and 3.5-fold) compared to control (Figure 5B). Inversely, the AF production was doubled and tripled in the infected grains with the exposed fungi to TCDD at 10 and 50 ng mL^–1^ compared to control (Figure 5B). These data indicate that the exposure of *A. flavus* to dioxin could likely boost its aflatoxicogenicity.

## Discussion

Aflatoxins are globally considered the most perilous food contaminants with proven toxicological, economic and social impacts. The fact that these toxins are mainly produced by certain fungal species that are known by their remarkable adaptive capabilities with various environmental niches, makes their management an effective challenge (Weckbach and Marth, 1977; Mehl et al., 2012). In this context, the focus on the biological impacts of environmental factors on the fungal growth, and development in general and its aflatoxicogenicity in particular, is a decisive rule for globally governing the contamination with aflatoxins in a sustainable minded manner. In line with this, many ecological studies have demonstrated the biological feedback of environments on the aggressivity/aflatoxicogenicity of AF-producing fungi (Gilbert et al., 2017; Medina et al., 2017). Unexpectedly, despite the ultimate connection of AF-producing fungi with soil, the biological impacts of soil contaminants, more particularly the POPs, are still obscure.

In light of this, the current study shows that the *in vitro* exposure of *A. flavus* to dioxin results in a phenotype typified by a peculiarly reducing in fungal vegetative growth and a noteworthy tendency to conidiation. First, in the absence of comparative data in fungi, the limitation in fungal mycelial growth caused by TCDD could be supported by a similar phenomenon in the experimental animals exposed to TCDD. A characteristic feature of acute exposure to dioxin is a dramatic decline in body weight, a phenomenon known as the wasting syndrome (Tuomisto et al., 1995; Tsujimoto et al., 2013; Hutin et al., 2018). With surprising similarity, a significant reduction in the fresh weight of leaves and seeds was also reported for plants that were experimentally exposed to TCDD (Zhang et al., 2012; Hanano et al., 2014a, 2018b). Beyond the wasting of weight, the dioxin-exposed organisms tended to implant a specific profile of cellular fatty acids, which leads to a puzzling and dramatic perturbation of the regulatory systems for energy balance (Hanano et al., 2018b,c). Moreover, the TCDD-induced phenotype of *A. flavus* was characterized by a remarkable ability to sporulate. Indeed, there are multiple lines of genetic, molecular and biochemical evidence that confirm the presence of a biological link between the fungal sporulation and the accumulation of secondary metabolites (SM) and more particularly the AF, where their production was also enhanced upon exposure to TCDD. In this context, earlier reports have shown that the environmental conditions required for sporulation and the accumulation of SM were often similar and were more strict than those for vegetative growth (Sekiguchi and Gaucher, 1977; Guzman-De-Pena and Ruiz-Herrera, 1997). This included, but was not limited to, temperature (Espeso et al., 1993), air-surface interface (Guzman-De-Pena and Ruiz-Herrera, 1997), medium pH (Buchanan and Ayres, 1975), nature of nutrients (Keller et al., 1997) and certain compounds present in seeds commonly infected by *Aspergillus* species (Goodrich-Tanrikulu et al., 1995; Burow et al., 1997; Calvo et al., 1999; Zeringue, 2000). In a more evolved manner, it was suggested that certain SMs act as sporogenic factors. For example, several lines of genetic evidence have shown that the accumulation of natural pigments in fungal tissues, such as melanin, is associated with fungal sporulation (Kawamura et al., 1999). Of particular interest, the relationship between fungal sporulation and mycotoxin biosynthesis has been corroborated for AF biosynthesis in *A. flavus* and *A. parasiticus* as well as for sterigmatocystin (ST) biosynthesis in *Aspergillus nidulans*. The non-producing AF/ST mutants that had a deletion in *aflR*, a gene encoding a regulatory protein that modulates the expression of AF/ST-biosynthesis cluster genes, failed in the formation of spores in all three *Aspergillus* sp. (Trail et al., 1995). Inversely, the overexpression of *aflR* enhances the production of AF and the formation of spores (Yu et al., 1996), and this is in line with our data showing an increasing level in *aflR* transcripts in fungal tissues after exposure to dioxin. Furthermore, the fluffy mutants of *A. nidulans* and *A. flavus* that have a defective G-protein signaling pathway resulted in an aconidial, aflatoxin-null phenotype (Calvo et al., 2002; Affeldt et al., 2012). More recently, the nucleoside diphosphate kinase (AfNDK), which was newly characterized in *A. flavus*, regulates spore and sclerotia development and is involved in plant infection (Wang et al., 2019). Additionally, the HosA, a new histone deacetylase recently identified in *A. flavus*, plays a determinant role in growth, development and AF biosynthesis (Lan et al., 2019).

Substantial dioxin-induced overproduction of AF can be an expected result of the increasing level of ROS in the fungal tissues upon their exposure to TCDD. In line with this, dioxins have a proven effect in inducing the accumulation of ROS in exposed animals (Bentli et al., 2016; Liu et al., 2016) and plants (Hanano et al., 2014a), suggesting a similar effect in fungi. Otherwise, the enhanced level of ROS was effectively accompanied with a significant stimulation of SOD and CAT activities, the two main scavengers of O_2.–_ and H_2_O_2_, respectively. This could be viewed as a part of the complex anti-oxidative defense system used by the organism to minimize the oxidative damage initiated by the ROS access. The activities of both enzymes were stimulated in plant tissues upon exposure to dioxin, polyaromatic hydrocarbons and some pesticides (Liu et al., 2009; Zhang et al., 2012; Hanano et al., 2014a).

In respect to the induction of AfPXG by TCDD, the expression of this caleosin is modulated by various biotic and abiotic stresses in several fungal species e.g., *Aspergillus oryzae* (Machida et al., 2005; Akao et al., 2007), *Ustilago maydis* (Tollot et al., 2016), and *Erysiphe necator* (Wakefield et al., 2011). Furthermore, the TCDD-induced expression of AfPXG led to an increase in the accumulation and the stability of LDs in fungal tissues. This result can be supported by recent findings showing that the heterologous expression of AfPXG in yeast led to a massive accumulation of stable LDs (Hanano et al., 2015). Likewise, the AfPXG-overexpressed line of *A. flavus* accumulated more LDs than the wild type (Hanano et al., 2018a), and this was synchronized with an elevated level in AF secretion. It is likely that the expression of AfPXG could be coordinated by TCDD at two levels;

(i) At the level of AfPXG enzymatic activity, it is now well recognized that AfPXG experimentally catalyzes the reduction of fatty acid hydroperoxides (FA-OOH) into their corresponding alcohols (AF-OH) as the plant caleosins/peroxygenases typically do (Hanano et al., 2006, 2016b). The biological impacts of this activity, characterized as the newest branch of the oxylipins biosynthesis pathway, have been interestingly underlined in plants (Blee et al., 2014; Charuchinda et al., 2015) but less in fungi (Fan et al., 2015). For example, it was documented that the endogenous treatment of *Aspergillus* with plant-originated FA-OOH represses AF biosynthesis and lengthens the time during which the AF gene transcripts accumulate (Burow et al., 1997). More recently, the knockout of AfPXG in *A. flavus* resulted in a considerable accumulation of FA-OOH in fungal tissues that have been accompanied with developmental anomalies and lowering in AF production (Hanano et al., 2018a).
(ii) At the level of AfPXG’s structural role in LDs assembly, the caleosins encoding genes are present in the vast majority of publicly available fungal genomic sequences including all *Aspergillus* spp., and they contain at least one copy of a highly conserved transmembrane domain enabling the caleosin proteins to be targeted in the monolayer membrane of LDs (Murphy, 2012; Hanano et al., 2015; Rahman et al., 2018a, b). The structural role of fungal caleosins in impacting the assembly and the stability of LDs is well demonstrated (Froissard et al., 2009; Jamme et al., 2013; Hanano et al., 2015), and this is in line with our results indicating that the TCDD-exposed fungi expressed more AfPXG and therefore accumulated more of LDs than the control. Otherwise, similar inductions of certain isoforms of caleosins were also reported in plants that have been experimentally exposed to TCDD (Hanano et al., 2016b, 2018b,c).

In connection with the TCDD-induced hyperaflatoxicogenicity, the implication of LDs in trafficking and exporting of AF has been recently reported (Hanano et al., 2018a), where the increasing number of LDs in the AfPXG-overexpressed line of *A. flavus* showed an increasing capability to sequestrate the AF and this was positively correlated with a high secretion ration of AF into the medium. In line with this, the role of LDs as transient repositories of lipophilic and hydrophilic compounds, from small molecules such as DNA, signal molecules, SM to large proteins, is well established (Chang et al., 2015; Gao and Goodman, 2015; Kory et al., 2016; Schuldiner and Bohnert, 2017). Taken together, our data suggest that the dioxin-induced hyperaflatoxicogenic phenotype of *A. flavus* is likely mediated by the caleosin/peroxygenase AfPXG. This could be particularly supported by using the *AfPXG-*silencing strain of *A. flavus*, where the typical TCDD-induced phenotype observed in the WT was completely abolished in the *AfPXG-*silencing strain. Finally, we found that the TCDD-exposed *A. flavus* had increased virulence against the grains of maize in terms of fungal invasion, sporulation and AF production. These observations suggest that contamination of soil with dioxin can influence the invasion capacity of soil-dwelling *A. flavus*, more particularly the fungal spores, empowering its aflatoxicogenicity. Similar worrying scenarios were suggested in response to possible adverse consequences of climate change (Medina et al., 2014; Assuncao et al., 2018).

## Conclusion

Our study sheds light, for the first time, on the possible biological feedback of persistent environmental pollutants, notably the dioxins on the aggressivity of AF-producing fungi in terms of their ability to infect and contaminate the crops. The production of aflatoxins and their subsequent prevalence in agricultural commodities are affected by environmental changes including persistent pollutants such as dioxins, which are increasingly recognized as direct contaminants resulting from the forests burning. Our data showed that the *in vitro* exposure of *A. flavus* to dioxin considerably increased its virulence in term of sporulation and aflatoxicogenicity. We suggest that this hyper-aflatoxicogenic phenotype is mediated by the caleosin/peroxygenase AfPXG that has proven functions in controlling the biosynthesis and trafficking of aflatoxins in *A. flavus*. This study highlights one unpredicted consequence of climate change that is relevant to the food safety. Our observations suggest that global food safety could be impacted by climate change, and this will possibly present new challenges in the near future to combat climate change in a global manner as well as to preserve, and even more to reinforce, the global regulations of food safety.

## Data Availability Statement

All datasets generated for this study are included in the manuscript/supplementary files.

## Author Contributions

AH led the work, designed all experiments in biochemistry and molecular biology, and wrote the manuscript. MS and IA carried out all experimental work. All authors read and approved the final manuscript.

## Conflict of Interest

The authors declare that the research was conducted in the absence of any commercial or financial relationships that could be construed as a potential conflict of interest.
